# Contraindications of sentinel lymph node biopsy: Áre there any really?

**DOI:** 10.1186/1477-7819-5-10

**Published:** 2007-01-29

**Authors:** George M Filippakis, George Zografos

**Affiliations:** 1 General Surgery Unit, Breast and Endocrine Department, St.Mary's Hospital, NHS Trust London W2 1NY, UK; 2 A' Propaedeutic Surgical Department, Hippokration General Hospital, Athens, Greece

## Abstract

**Background:**

One of the most exciting and talked about new surgical techniques in breast cancer surgery is the sentinel lymph node biopsy. It is an alternative procedure to standard axillary lymph node dissection, which makes possible less invasive surgery and side effects for patients with early breast cancer that wouldn't benefit further from axillary lymph node clearance. Sentinel lymph node biopsy helps to accurately evaluate the status of the axilla and the extent of disease, but also determines appropriate adjuvant treatment and long-term follow-up. However, like all surgical procedures, the sentinel lymph node biopsy is not appropriate for each and every patient.

**Methods:**

In this article we review the absolute and relative contraindications of the procedure in respect to clinically positive axilla, neoadjuvant therapy, tumor size, multicentric and multifocal disease, in situ carcinoma, pregnancy, age, body-mass index, allergies to dye and/or radio colloid and prior breast and/or axillary surgery.

**Results:**

Certain conditions involving host factors and tumor biologic characteristics may have a negative impact on the success rate and accuracy of the procedure. The overall fraction of patients unsuitable or with multiple risk factors that may compromise the success of the sentinel lymph node biopsy, is very small. Nevertheless, these patients need to be successfully identified, appropriately advised and cautioned, and so do the surgeons that perform the procedure.

**Conclusion:**

When performed by an experienced multi-disciplinary team, the SLNB is a highly effective and accurate alternative to standard level I and II axillary clearance in the vast majority of patients with early breast cancer.

## Background

The pathologic status of the axillary lymph nodes remains the most important prognostic indicator for patients with breast cancer and a major determinant of adjuvant treatment. Sentinel lymph node biopsy (SLNB) has become a widely accepted evaluation and staging procedure for the axilla in patients with breast cancer, and this is mainly due to the minimal morbidity and the high degree of histological accuracy it provides. When performed by an experienced multi-disciplinary team, the SLNB is a highly effective and accurate alternative to standard level I and II axillary clearance. To ensure and maintain the high accuracy and low false-negative rate of the SLNB procedure, several selection criteria and relative contraindications for the procedure have been reported, together with few safety issues. These contraindications include host factors such as disturbed lymphatics due to prior breast and axillary biopsy and/or surgery, age, body-mass index, pregnancy, and tumor biologic characteristics such as tumor size, multifocal or multicentric disease and histological type (in situ carcinomas). Furthermore, safety issues like radiation levels to patient (especially during pregnancy) and medical stuff as well as storage and disposal of radioactive waste, still remain debatable issues. Certain authors suggest that when present, these factors may affect negatively the accuracy of the SLNB, resulting in failure of the procedure or higher than acceptable false negative results. We review the literature in respect to each and every one of those factors and discuss the impact they may have on the degree of accuracy and efficacy of the procedure.

## Absolute contraindications for SLNB

### Clinically positive (N1) axilla

The absolute contraindications for the SLNB are quite clear and straightforward. When there is clinically suspicious axillary lymphadenopathy or when fine needle aspiration cytology and/or core biopsy of palpable axillary lymph nodes confirm tumor infiltration, the procedure is contraindicated. According to the American Society of Clinical Oncology Guideline Recommendations for Sentinel Lymph Node Biopsy in 2005 and the 2001 Proceedings of the Consensus Conference on the Role of SLNB in Carcinoma of the Breast in Philadelphia [1,2], patients with clinically positive axilla (N1) are not candidates for the procedure. It has been suggested that in such cases, the path of the dye or the radio-colloid agent may be blocked from tumor cells infiltrating the lymph vessels. This could prevent the identification of the true sentinel node(s) and result in failure of the procedure or false negative results. Sentinel lymph node biopsy is best reserved for breast cancer patients with clinically negative axillary nodes [3], except perhaps in the setting of clinical trials. However, for those patients with suspicious axillary findings, preoperative ultrasound and fine needle aspiration (FNA) cytology can provide further information on the status of the nodes and help surgical planning. Approximately, 40% of patients with positive axillary nodes can be diagnosed preoperatively with ultrasonography and FNA cytology alone [4]. In node positive patients a standard level I and II axillary clearance is indicated and SLNB is not performed. In patients with clinically suspicious nodes and negative FNA cytology, SLNB can be performed if bearing in mind that any clinically suspicious palpable axillary nodes encountered during the procedure should be excised and examined, regardless of whether they take up dye or radio-colloid, even if they are not the true sentinel node(s) [1,2].

### Allergy to blue dye and radio-colloid

History of allergy to the radio-colloid and/or blue dye should be considered contraindication to the use of that specific agent. There has been observed no cross-reactivity between these two agents. In the United States the most commonly used agents are technetium sulphur colloid and isosulfan blue dye, whereas in Europe technetium labelled albumin and patent blue. Blue dyes used for lymphatic mapping in sentinel lymph node biopsy cause intraoperative anaphylactic reactions in up to 2.7% of patients [5,6]. These agents include patent blue, isosulfan blue and methylene blue. The sodium salt of patent blue is also called sulfan blue, food blue 3, patent blue VF, and acid blue 1. Isosulfan blue is a structural isomer of patent blue (not patent blue V) and is known under the trade name lymphazurine. Patent blue V has a slightly different chemical structure and can also be found under the name patent blue violet, food blue 5, acid blue 3, and disulfine blue. It is also known as E 131 and is still on the market as a food colorant. However, because of the close structural relationship of these vital dyes, cross-reactivity may indeed exist [5]. Methylene blue (anhydrous methylene blue or methylene blue trihydrate), is only approved for intravenous administration for the treatment of methemoglobinemia and hemolysis because it may cause necrosis on subcutaneous administration. Methylene blue is structurally not related to isosulfan blue or patent blue V and, therefore, cross-reactivity is not to be expected. However, according to certain authors, since methylene blue has no sulfonic acid groups it does not bind to plasma proteins and might not be taken up by lymph, diffusing directly into blood capillaries [6]. For the detection of hypersensitivity (mainly type I) to blue dyes, intracutaneous tests are valuable tools. In most cases, sensitization exists without known previous exposure, and this may be due to the widespread use of such dyes in objects of everyday life. Allergy to cosmetics containing blue dye consist a relative contraindication in the use of the blue dye, but previous allergic reactions to sulfa or sulphur do not predict reaction to the dye [1]. At least two deaths have been described from anaphylactic reactions to the blue dye and while adverse reactions in general are very rare (1–3%), they can be extremely severe. Preoperative antiallergic medication use does not prevent anaphylactic reactions but greatly reduces their severity [7]. Patients with sulfa allergies and prior exposure to lymphatic mapping do not generally demonstrate an increased incidence of adverse drug reactions [8].

Combined use of blue dye and radio-colloid is considered to be a very effective technique and some authors suggest an overall added value for the sentinel lymph node biopsy [9,10], especially when young surgeons are just learning how to perform the procedure. Whether both techniques together are superior to either alone is still debatable. In a recent randomized clinical trial comparing blue dye alone with combined dye and isotope technique for sentinel lymph node biopsy in breast cancer, combined mapping was superior to blue dye alone in identification of the sentinel node(s), but accuracy and false-negative rates were similar [11]. Other reports suggest that with the increasing success of isotope mapping, the marginal benefit of blue dye utilization (when the sentinel lymph node is identified by blue dye alone) is small and declines further with experience (from 9% – 3%) [12], making the application of radio-colloid only, the preferred method for SLNB [13]. Hand-held gamma probes are nowadays smaller and more manoeuvrable, with better shielding for directional detection of gamma rays, making the method even more applicable. Furthermore, the reported high identification rates of the sentinel lymph node(s) with the use of radio-colloid only, make the isotope radio guided method very sensitive and extremely successful, avoiding all possible blue dye anaphylactic reactions in allergic patients.

## Relative contraindications

Previous breast surgery may disrupt the lymphatics from the tumor site to the axilla and this may lead to higher false negative results or failed procedures [14]. This seems to be true for extensive breast surgery such as reduction mammoplasty or quadradenctomy but not for more conservative surgery or biopsy procedures.

### Prior biopsy

Biopsy methods of a suspicious breast lump include fine needle aspiration cytology, core or true cut biopsy, vacuum assisted stereotactic biopsy and even more invasive procedures such as incisional or excisional biopsy. Many of the important prospective, large-series, reports on SLNB excluded patients with previous excisional biopsy [15-17] and/or previous axillary surgery [18], making the true impact of prior breast biopsy rather uncertain. Some studies have suggested that SLNB for breast cancer may be less accurate after excisional biopsy of the primary tumor compared with core needle biopsy or fine-needle aspiration biopsy. The main concern is that large-volume excisional biopsy results in subsequent disruption of breast lymphatics, decreasing the likelihood of successful lymphatic mapping. Therefore, any nodes removed after an excisional biopsy may not actually be an accurate reflection of lymphatic drainage from the site of the primary tumor. High failed sentinel lymph node identification rates (up to a 7-fold increase) after excisional biopsy and a significantly higher lymphatic mapping failure rate was reported by certain authors [18,19]. Feldman et al. [14], in their study suggested that false-negative results in SLNB were increased only in patients who had prior excisional biopsies, but also reported increased accuracy for the procedure once the protocols were altered with increased number and volume of injections. In all three studies, only radioactive colloid was used for lymphatic mapping via peritumoral injection.

However, ample up to date data, suggests that prior breast biopsy, including excisional biopsy, does not affect the success or the accuracy of the procedure [20-24]. Haigh et al., [20] reported that SLNB was highly successful in breast cancer patients regardless of biopsy method (stereotactic core biopsy, fine-needle aspiration, or excisional biopsy), excision volume, and the interval between the biopsy and the SLNB procedure. In addition, Miner et al., [25] also demonstrated that SLNB is comparably successful regardless the extent of the prior biopsy. The University of Louisville Breast Cancer Sentinel Lymph Node Study, in a prospective multi-institutional study reported that excisional biopsy does not significantly affect the accuracy of the SLNB, nor does changes the type of definitive surgical procedure [26]. Data from the European Institute of Oncology is in accordance, suggesting that SLNB accuracy after a previous breast biopsy is comparable with the results obtained in validation studies [24]. False negative results [22,23] and regional recurrence rates [24] seem to be similar after fine needle aspiration or excisional biopsy as other multi-institutional studies confirm. Results from the John Wayne Cancer Institute, demonstrated that SLNB was accurate and successful regardless of the biopsy procedure previously performed or the volume of the tissue removed [20]. Furthermore, it was shown that the size of the tumor, interval from prior biopsy and the tumor location did not significantly alter the procedure, with false negative rates for the entire series of 3,2%. Therefore, it seems that any type of previous breast biopsy (including excisional biopsy) does not affect the accuracy or success rate of SLNB [2,27-29]. It is however, warranted that injection of radio colloid or dye directly into the biopsy cavity should be avoided since it may cause spillage and inaccurate lymphatic mapping.

Lymphoscintigraphy (LS) is often a very useful tool to combine with SLNB, especially in patients who have already undergone excisional biopsy. Recent reports suggest that the sensitivity and visualization rates for lymphoscintigraphy seem to be unaffected by prior excisional biopsy [21]. In two prospective studies using completion axillary dissection, the accuracy, sensitivity and predictive value of intraoperative lymphatic mapping and the SLNB was unchanged regardless of previous excisional biopsy, when a sentinel node was visualized on the preoperative lymphoscintigram [20,30]. Lymphoscintigraphy in SLNB can provide an accurate map of the pattern of lymphatic drainage from the primary tumor site and successfully identify the sentinel node(s). This is very important in cases of non-axillary drainage of the tumor or disrupted lymphatics. The implications of such drainage to unpredicted lymph nodes are indeed profound. In such cases the sentinel node(s) may be found in other, less frequent, sites that include the internal mammary chain, the subclavicular area, the contralateral axillary basin, the interpectoral lymph nodes or the breast parenchyma. Therefore, imaging is essential for preoperative localization of possible non-axillary sentinel lymph node(s). Lymphoscintigraphy with direct visualization of the sentinel node(s) may reduce the failure rate of SLNB and even lower the small, but significant, percentage of false negative rates that result from the existence of alternative (non-axillary) lymphatic drainage pathways [31]. Visualization rates for LS are reported between 76%-95% using 99mTc-labeled small particle radio-colloids and high-resolution collimators. Non-visualization of the lymphatic pathways and/or sentinel node(s) may be due to either technical errors, such as inadequate utilization of radio-colloid, improper injection site and inappropriate timing, or to true lymphatic blocking from cancer cells. Moreover, an extensively damaged lymphatic network from previous surgery may impede radiopharmaceutical migration, causing non visualization of the tumour's sentinel node(s). The use of a sufficient amount of radioactivity (at least 100 MBq) is recommended for lymphatic mapping in breast cancer, especially in obese and/or elderly women. It has been shown that delayed imaging and re-injection of the radioactive tracer can increase the visualisation rate. In a multivariate regression analysis [32], scintigraphic non-visualisation was independently associated with increased patient age, decreased tracer dose and increased number of tumour-positive lymph nodes. Other reports seem to concur that patients with tumor positive nodes have increased risk of LS failure and report failure rates of up to 50% when more than four nodes are involved with metastatic disease. However, non-visualised sentinel node(s) during lymphoscintigraphy can be identified intraoperative in more than half of the patients, using a hand held gamma probe and/or blue dye. In the rest patients, palpation of the axilla during surgery and axillary node sampling seems to be a safe alternative. For this reasons non visualization of the sentinel node during LS should not be considered a contraindication to SLNB. Furthermore, certain authors suggest that lymphoscintigraphy findings do not enhance the success of intraoperative identification of the sentinel node nor alter the postoperative management of patients with early stage breast cancer and thus, should not be considered a routine procedure [33].

### Previous breast and axillary surgery

An increasing proportion of patients with breast cancer choose to undergo breast conserving therapy (BCT) and 10–15% of them will develop local or regional recurrent disease within 10 years of follow-up [34,35]. Similarly, the same proportion of local relapses is seen in patients with ductal carcinoma in situ (DCIS) treated with BCT and radiotherapy [36,37]. This is the main reason why the already proven efficacy of the SLNB in primary breast disease has led to a new interest for the procedure in the re-operative setting. Although previous breast surgery was considered a contra-indication for SLNB, today this notion is being re-evaluated. The minimum requirement for a successful SLNB is the presence of a patent lymphatic pathway between the tumor (either primary or recurrent) and the sentinel node(s). Disrupted lymphatics may as well be reformed soon after the initial operation or be re-diverted in a different lymphatic route and an unobstructed lymphatic system is assumed if no postoperative lymphedema is present. According to many authors [1,2,27-29], any kind of biopsy for breast cancer as well as non extensive breast surgery, is not a contraindication to SLNB and does not vitiate its success. However, more radical breast surgery such as reduction mammoplasty, augmentation mammoplasty through an axillary incision and quadradenctomy may be related to higher false negative rates and inability to locate the sentinel node(s). Again, the most important anatomic consideration in mind is an intact lymphatic system and thus, if the tumor is located in an intact quadrant of the breast, even a recent reduction procedure is unlikely to affect the efficacy of the SLNB [1]. Currently there are no data to discourage the use of SLNB in breast cancer patients who undergo immediate reconstruction or who previously had augmentation or reduction mammoplasty [38,39]. Lymphatics from the lateral and upper quadrants of the breast to the axilla are usually not damaged after breast reduction surgery or cosmetic breast implants in the sub-mammary or sub-pectoral position, especially if the surgery was performed more than 6–12 months previously [2]. More studies are needed to elucidate this matter, but if the SLNB is performed in these settings, it may be safer to be combined with preoperative lymphoscintigraphy to evaluate lymphatic routing.

Similarly, SLNB after axillary surgery has not been widely evaluated and most reports exclude patients with prior axillary dissection. However, few published studies report that prior axillary dissection seems to make the procedure less accurate. Port et al., reported that repeat SLNB failed in 25% of patients compared with less than 5% among women who had not previous axillary surgery [40]. On the other hand, a study from the Memorial Sloan Kettering Cancer Centre showed that re-operative SLNB after previous axillary lymph node dissection (ALND) is feasible in selected breast cancer patients and is more likely to succeed when fewer that 10 nodes were removed during the earlier procedure [41]. In this study, the identification rate for the SLNB in patients with primary breast carcinoma was between 94% and 97% with a false negative rate of 4–5%. In the re-operative setting identification rate fall to 87% in patents with 10 or less axillary lymph nodes previously removed and to 44% if more than 10 nodes were previously excised. With increasing number of nodes previously removed, re-operative SLNB was less successful. Recent data from the European Institute of Oncology is in accordance with these findings and suggests that SLNB after a previous breast or axillary operation is technically feasible and effective, but in selected breast cancer patients with local recurrence [41]. For the 10–15% of breast cancer patients who develop local or regional relapse, SLNB has exhilarating potential, but a larger patient population and a longer follow-up are necessary to confirm these exciting preliminary multi-institutional findings. False negative rates after prior axillary dissection are very difficult to determine and require long term follow-up, since a previous axillary lymph node dissection removing the majority of axillary nodes will leave only the sentinel node(s) remaining and so a back-up axillary clearance is impossible to perform.

### Advanced disease and neoadjuvant chemotherapy

Most patients with locally advanced breast cancer have large tumors and a high incidence of axillary node involvement, for which they may be offered neoadjuvant chemotherapy. However, a significant proportion of these patients (40%-48%) will not harbour metastatic disease in the axilla and thus may be spared an unnecessary ALND. Neoadjuvant chemotherapy (NC) was initially used to down-stage what is considered inoperable disease. Under those circumstances, tumor response implied also response in regional or even distant metastases [42]. In addition, neoadjuvant chemotherapy has been applied with success in locally advance disease. Recently, NC is being offered to patients with smaller tumors (stage II disease, with tumors larger than 3 cm), since trials have demonstrated that breast conservation treatment following neoadjuvant radiotherapy is as safe in terms of local-regional control and overall survival, as mastectomy [43,44]. SLNB has been used so far, to a very limited degree, in selected breast cancer patients with locally advanced degree receiving neoadjuvant chemotherapy and many authors consider that it should not be performed outside a clinical trial [1]. The success rate of the procedure may be lower and false negative rates higher due to histological changes in the breast and the draining lymphatics caused by the chemotherapeutic agents [45], and in the possible regression of the disease in the sentinel node(s) but not elsewhere in the axilla. It has been suggested that, alterations in the anatomy of the lymphatics draining the tumor could interfere with the lymphotropic agents and prevent their unobstructed transit to the sentinel node(s). However, if this was true, a difference in the site and number of sentinel nodes might be expected as well as altered absorption of radio-colloid agent resulting in either higher or lower sentinel node radioactivity counts. Recent studies have failed to prove this theory, finding no differences in the number, location or radioactivity status of the sentinel node(s) in patients receiving neoadjuvant therapy [46]. Lymphatic mapping has demonstrated that lymphatics in the breast are regional and not point specific, covering wide areas of the breast and this conclusion further rationalizes the practice of the SLNB. Moreover, recent data suggest a role for SLNB in the subgroup of patients treated with neoadjuvant chemotherapy and clinically or ultrasound negative axillary nodes (N0) at the end of the treatment. Response rates to neoadjuvant chemotherapy range from 65% to 90% [47], and the majority of patients do achieve significant regression of both the primary tumor and the axilla metastases. Therefore, it is reasonable to re-evaluate the role of the SLNB in this patient subgroup. In the largest report to date, updates data from the NSABP B-27 prospective randomized trial evaluating the efficacy of preoperative versus postoperative docetaxel following preoperative doxorubicin/cyclophosamide chemotherapy [48], an identification rate for the sentinel node of 85% and false negative rates of 12% were reported. Data from smaller studies are in agreement, reporting identification rates between 85% and 94% [49,50] and false negative rates of as low as 0% to as high as an unacceptable 33% [51-56]. It seems that these preliminary reports suggest a potent role for SLNB in locally advanced cancer and patients receiving induction chemotherapy (except in inflammatory cancer), but clearly, more trials including large number of patients need to clarify the efficacy of SLNB in the neoadjuvant setting. Moreover, there is no current data on the long-run regional recurrence rate in these patients, and since neoadjuvant treatment may eradicate metastatic foci in the axillary lymph nodes, the long term clinical and prognostic significance of negative findings after the SLNB is not clear yet.

### Tumour size

It is well documented that the incidence of axillary tumour metastasis increases in respect with tumour size [57]. Few years ago when the SLNB was first introduced, the majority of surgeons reserved the procedure for breast cancer patients with tumor size less than 2 cm. Even today, the accuracy of SLNB had not been verified in larger cancers, but it is becoming apparent that SLNB is technically feasible and highly accurate even in larger (T2 and T3) tumors. About 30% of patients with breast tumors larger than 4 cm have no axillary lymph mode metastasis and therefore do not benefit from ALND. In a small prospective study of SLNB followed by complete ALND for breast cancers larger than 4 cm, both the identification rate and the sensitivity of the procedure was equivalent to those patients with smaller tumors [58]. Several other studies concur, indicating no significant difference in both the identification and false-negative rate in T3 versus T1 tumors [59-62]. Therefore tumor size should not preclude the use of the SLNB in those patients otherwise candidates for the procedure. Axillary lymph node dissection can be avoided in nearly one third of patients with larger tumours by focused examination of the sentinel lymph node. However, the axilla must be always thoroughly palpated during the SLNB procedure and any suspicious lymph nodes should be removed.

### Multicentric and multifocal disease

The incidence of multicentric (MC) and multifocal (MF) disease in breast cancer ranges between 13% and 65% [63]. By definition, the term multicentric disease describes two or more carcinomas arising in separate quadrants of the breast, or tumors more than 2–5 cm apart [64]. The term multifocal disease refers to the presence of separate foci of carcinoma in the same quadrant of the breast. The presence of MC or MF in invasive breast cancer was considered a relative contraindication for the SLNB, since there are suspicions that the multiple tumors might involve more than one dominant lymphatic trunk draining to the regional axillary lymph nodes. Multiple lymphatic trunks might drain to different sentinel lymph node(s) and may be overlooked. However, with the use of lymphatic mapping and the increased experience in SLNB, there is now increasing evidence-based support of the theory that the lymphatics of the mammary gland drain through a few common afferent lymphatic trunks to specific axillary sentinel lymph nodes, regardless of the tumor location [65,66]. Evidence obtained the past few years about the functional anatomy of the lymphatic drainage of the breast supports the theory that all quadrants of the breast drain into same lymph node(s) [67,68]. In addition, there is a deep and a superficial lymphatic system with a subareolar plexus that drains the axillary lymph nodes via one or two main lymphatic vessels [69]. According to the Proceedings of the Consensus Conference on the Role of Sentinel Lymph Node Biopsy in Carcinoma of the Breast (2001), SLNB is not recommended for women with multicentric disease outside the setting of research protocols. On the other hand, the panel concluded that multifocal disease is not a contraindication for the procedure, when the tumors diameter is less than 3 – 5 cm [1]. The American Society of Clinical Oncology Guidelines Recommendations for Sentinel Lymph Node Biopsy (2005), considers the procedure acceptable for MC-MF breast cancer, but notes that evidence is limited to small scale trials [2]. A recent multi-institutional trial presented at the 55^th ^Annual Cancer Symposium of the Society of Surgical Oncology, reported no significant differences in the identification rate and false negative rates between patients with MC and MF disease in comparison with single site breast cancer patients [70], and since then a few other studies have validated these findings [71,72]. Several small non-randomized studies also reported that the accuracy and efficacy of SLNB by subareolar or intradermal injection of tracers was similar to that of women with unifocal disease [71,73-75]. Reported identification rates in MC and MF range from 90%-97% [70,76] and false negative rates from 0% to 8% [71]. In their series, with detection rate of 97% and false-negative rate of 8%, the group at the Memorial Sloan-Kettering Cancer Centre concluded that the accuracy of SLNB was comparable to unicentric lesions and the Little Rock group concurred [75]. Studies with larger patient populations and long term follow-up are necessary to prove the efficacy of SLNB in the MC-MF setting, but preliminary results suggest that the procedure may be an accurate alternative to ALND in patients with clinically negative axilla and multicentral or multifocal breast cancer [76].

### Ductal Carcinoma in-Situ

Ductal carcinoma in-situ (DCIS) has become nowadays a common finding in patients with screen detected breast cancer and represents approximately 20% of all breast malignancies. By definition DCIS has no metastatic potential while in the in situ phase, however extensive DCIS may harbour invasive component that can potentially spread to the axillary lymph nodes. This invasive pattern may be missed in up to 5% to 20% of patients undergoing core biopsy, because it may represent only a small proportion of the overall tumor [77]. Although node metastasis in patients with true DCIS are very uncommon (0%-3%), sentinel node metastasis have been detected in 13%-20% of extensive tumors with high nuclear grade or areas of necrosis [78,79]. Other high risk features of DCIS include a palpable or mammographic mass with pluricentric microcalcifications, comedo type, multicentric disease. and usually those patients are treated by mastectomy. A very extensive and careful histological examination of the tumour in DCIS is compulsory to exclude any micro-invasive foci. Handling of the SLNB specimen by the pathologist is crucial and detailed definitions of metastasis have become important, especially since immunohistochemistry techniques have been used used [80]. The use of immunohistochemistry (IHC) to detect keratin proteins will reveal metastatic breast carcinoma in about 18% of axillary or sentinel lymph nodes that appear negative on routine stains [81]. To date evidence suggests a significantly poorer prognosis in patients with such occult metastases, although data from large prospective studies are lacking. The term "micro-metastasis" refers to a cohesive cluster of malignant cells larger than 0.2 mm but smaller than 2 mm in diameter. "Submicroscopic metastases" best describes small cell clusters or individual tumor cells less than 0.2 mm in diameter found by immunohistochemistry techniques. The need for completion axillary node dissection in these cases is still controversial. For years there has been speculation that micrometastases in axillary lymph nodes were clinically insignificant [82], however most of the recent studies have found a significantly poorer prognosis associated with micrometastases, suggesting that even such small metastases cannot be safely overlooked [81,83]. Some authors believe that micrometastases should be treated with completion ALND or with adjuvant radiation therapy [83-85]. On the other hand, no current evidence exist that submicroscopic metastases or individual tumors cells found within the sentinel lymph have an important prognostic significance [86], and therefore require no further treatment [83,87].

In conclusion, it seems that for patients with high risk DCIS for invasive component, SLNB is justified and therefore recommended as an efficient procedure to evaluate the status of the axilla [1,88,89]. On the other hand, in patients with low risk DCIS (such as true non-comedo DCIS) that is completely excised by surgery and with free margins of resection, SLNB should be avoided since not only it is unnecessary (due to the low possibility of metastatic involvement), but could also jeopardize a successive re-SLNB in case of invasive recurrence [90].

### Age and body mass index

Increased age and body mass index (BMI) have been linked to an increase incidence of SLNB failure [91,92]. This could have serious implications since a large proportion of patients with breast cancer are senior and/or overweight. Sentinel lymph node biopsy seems to be feasible regardless of the age of the patient but the identification rate of the procedure may be impacted by increasing age and BMI. Some authors speculate that anatomical changes in the breast of elderly and obese patients, with decrease in the density of the gland and increase of fatty tissue deposit, may result in decreased lymphatic flow and increased SLNB failure rates. Furthermore, the high content of subcutaneous and axillary adipose tissue makes palpation harder and the identification of the sentinel node more difficult, especially when only blue dye is utilized. Three multi-institutional studies found that the sentinel node was found less frequently in women older than 50 years of age [18,22,23], with reported identification rates of 87.6% versus 92.6% for younger patients [22], while other trials report a strong inverse relationship between increased BMI (>30) and identification rate for the SLNB [93]. The identification rate was 99% for patients with BMI < 20, 96.6% for BMI of 30 and 94.2% for BMI of 40. However, in all cases acceptable identification and false negative rates where achieved when the procedure was performed with use of both blue dye and radio-colloid. Dual-agent injection technique makes the procedure technical feasible and efficient in senior and/or overweight breast cancer patients and it seems that SLNB is a suitable and accurate alternative to routine axillary dissection for this particular patient population. However, surgeons performing the procedure should be aware that the radioactive counts might not be greater than 20 or 30 per node, and once again the surgical clinical judgment should guide all intraoperative decision making.

### Pregnancy

Breast cancer is the most common malignancy associated with pregnancy, with an incidence of 1 in 10,000 to 1 in 3,000. It is speculated that with the increase in delayed childbearing age, breast carcinoma diagnosed during pregnancy may become more frequent [94,95]. The role of SLNB in pregnant women with breast cancer is not clear. The radioactivity from the procedure might harm the foetus, plus the breast lymphatic pathways may be altered in pregnancy. Many authors suggest that since the risk of lymphatic mapping by blue dye and/or radiocolloid are still unknown, it is advised not to perform SLNB in pregnant women until more data is available [1,2]. The agents used for the identification of the sentinel node are blue dye and radio-colloid technetium. The blue dye might be blue de methylene, lymphazurin, isosulfan blue and patent blue. Blue dyes such as lymphazurine (a class C drug) have not been tested in pregnant animals or humans and thus should not be utilized in the pregnant or lactating patient. Radio-colloids include technetium sulphur colloid and technetium labelled albumin, which are considered safer agents. Recent data suggests that the radiation dose to the foetus is minimal, allowing reasonable consideration for the SLNB during pregnancy [2,96]. The amount of radioactivity utilized in SLNB is very low compared to standard radionuclide procedures. The radioactivity dose ranges from 250 μCi (1/40 of the bone scan dose) to 2 mCi and most of the injected radiocolloid stays at the injection site or moves to the sentinel node(s), both of which are excised during the procedure. Therefore, minute only amounts of radioactive colloid might reach the foetus with harmless effects. The total radiation exposure of the patient undergoing SLNB using 0.5 mCi of radiolabeled colloid is about the same as from a four exposure mammogram, at 0.4 mSv (1]. Recent studies claim that foetal exposure to radiation using radio-colloid agents such as technetium-99 m for lymphoscintigraphy and localization of the sentinel node is low and should not be considered a contraindication [97]. It might be so that the greater risk to the foetus is from the general anaesthesia, since foetal radiation doses are negligible and well below levels associated with risk concerns. Pandit-Taskar et al. [98], reported that the maximum estimated foetal exposure (with the worst case conservative assumptions) may be less than 3% of the Nuclear Regulatory Commission (NRC) monthly guidline of 0.5 mSv, and less than 0,3% of the NRC occupational exposure limits during gestation period, of 5 mSv. They suggest that lymphoscintigraphy and SLNB can be safely applied during pregnancy, as estimated radiation doses are not associated with significantly increased risk to the foetus. However, more experimental trials need to be performed to clear this issue and until that time, SLNB should not be offered to pregnant women under 30 weeks gestation, since this is the critical period for foetal organogenesis. Furthermore, since small quantities of the radioactive colloid can be excreted in breast milk, lactation should be avoided for a few days after the SLNB procedure.

### Radiation protection issues

The use of Technetium-99 m labeled colloids for lymphoscintigraphy and radio-guided surgery does not entail dangerous radiation burden to patients undergoing SLNB [99,100]. Furthermore, radioactivity levels to personnel handling contaminated materials is also negligible, since very low doses of radio-colloid are utilized during SLNB and at least 2 to 3 physical half lives elapse between tracer injection and the completion of the surgical procedure [101]. The cumulative doses involved in the procedure correspond at most to about 1% (mean absorbed dose) or about 10% (mean effective dose) of the annual dose limits for the general population. Radiation safety badges are not considered necessary for surgeons, nurses and pathologists. Radioactivity contamination in operating room materials is also minimal and does not require any special handling procedure. Letting radioactivity decay with time by storing the specimens for a few hours, is a sufficient precaution for pathologists handling the SLNB specimens. Special considerations for the waste radioactive materials are not required, but it is suggested that such waste materials are sealed and stored for decay at the nuclear department before disposal. If stored for 60 hours, which correlates to 10 technetium-99 m lives, the radioactive content of the waste falls 1000-fold. Transportation and disposal of decayed radioactive waste should proceed further according to local regulatory requirements.

### Surgeons experience and learning curve

SLNB is a multi-disciplinary effort involving surgical oncology, nuclear medicine and pathology. Each member of the team must rely on each other's contribution for the accurate and efficient performance of the procedure. It has been reported to exist a close correlation between the number of procedures performed by the team and the positive predictive value of the technique, with values ranging from 71% for experience of less than 40 procedures to 98% after more than 100 procedures [18,23]. The learning curve is considered to be completed after performing more than 50–60 procedures [102], and a success rate of more than 94% should be achieved. Full axillary lymph node dissection should be performed on all patients during the learning phase, irrespective of SLNB histology.

## Discussion

In breast cancer, hematogenous spread of tumor cells may precede or be coincident with lymphatic spread and so far regional lymphadenectomy has proven beneficial in terms of staging and loco-regional control of the disease, but has failed to produce an overall survival benefit. Furthermore, since about 70%-80% of ALND in breast cancer patients will reveal no metastatic disease, the benefits from regional lymphadenectomy, when compared with morbidity and cost, may be small. Sentinel lymph node biopsy is an alternative procedure to ALND which provides accurate information on the status of the regional lymphatics and converts elective or prophylactic axillary node dissection into beneficial therapeutic dissection [103]. Subsequently, only the breast cancer patients who truly profit from ALND will risk the high morbidity of this procedure and a great proportion of node negative patients will be spared an unnecessary operation. There are two important considerations that must be taken into account when certifying a multidisciplinary team for SLNB. First of all, the sentinel lymph node must be successfully identified in more than 95% of patients (failure rate < 5%) and secondly, the negative rate of the procedure should be less that 3–5%. Also, the fraction of patients with positive sentinel lymph node(s) should be 20%-30% in patients with T1a-b breast cancer and about 35% in patients with T1a-c cancer [101]. Higher rates are expected for larger tumors and more advanced disease. False negative results always harbor a risk for the patient undergoing SLNB. However, prevalence of clinically overt axillary metastases is less than 1% after a median follow-up of up to 57 months [16,104-106]. Currently there are randomized trials being conducted on the efficiency and efficacy of the SLNB that will provide answers to vital, unresolved issues such as the possible differences in survival and local-regional recurrence between standard axillary lymph node dissection and the SLNB.

## Conclusion

Like all surgical procedures, the sentinel node biopsy is not appropriate for each and every patient. Certain conditions involving not only host factors, but also tumor biologic characteristics may have a negative impact on the success rate of the procedure. However, the overall fraction of patients unsuitable or with many risk factors compromising the success of the SLNB is very small. Nevertheless, these patients need to be successfully identified, appropriately advised and cautioned, and so do the surgeons that perform SLNB. In conclusion, when performed by an experienced multi-disciplinary team, sentinel lymph node biopsy is a highly effective and accurate alternative to standard level I and II axillary clearance in the vast majority of patients with early breast cancer.

## Conflict of interest

The author(s) declare that they have no competing interests.

## Authors' contributions

**G.M.F **conceived and designed the study, carried out the literature review and drafted the manuscript.

**G.Z**. helped to draft the manuscript and revised the final version. All authors read and approved the final manuscript.
